# Vitamin D supplementation: upper limit for safety revisited?

**DOI:** 10.1007/s40520-020-01678-x

**Published:** 2020-08-28

**Authors:** René Rizzoli

**Affiliations:** Division of Bone Diseases, Geneva University Hospitals and Faculty of Medicine, 1211 Geneva 14, Switzerland

**Keywords:** Osteoporosis, Falls, Fracture, Bone health, Sarcopenia

## Abstract

Vitamin D overdosing includes hypercalcemia, hypercalciuria, and mineral deposits in soft tissues. A safety upper limit of 4000 IU/day, which is consistently accepted, has been challenged, since the risk of adverse events in other systems than calcium-phosphate homeostasis may depend not only on the dose, but on the outcome, the treatment regimen, and possibly the age, sex and vitamin D status. The therapeutic window of vitamin D supplementation may be narrower than hitherto recognized. The prevention and/or correction of vitamin D deficiency/insufficiency with 800–1000 IU/daily of vitamin D or 10 µg/day of calcifediol are safe. Because of their potential harm, larger doses given on the long term or in intermittent regimens should not be selected.

## Introduction

Vitamin D is an important regulator of calcium and phosphate homeostasis. Synthesized in the skin under the influence of UV light, vitamin D (cholecalciferol) undergoes a first hydroxylation in position 25 in the liver, leading to 25-hydroxyvitamin D (calcifediol), and a second one in position 1 in the kidney, leading to the active metabolite 1,25-dihydroxyvitamin D (calcitriol) [1]. The latter step is stimulated by PTH, IGF-I and by low calcium or phosphate intakes or concentrations. Calcitriol stimulates intestinal trans-epithelial transport of calcium and phosphate through both genomic and non-genomic mechanisms [1]. Calcitriol is a potent stimulator of bone resorption [2], by increasing the expression and production of RANKL by osteoblasts [3]. Vitamin D deficiency impairs hypertrophic cartilage and bone mineralization, leading to rickets in children and osteomalacia in adults. Vitamin D status is evaluated by the measurement of circulating 25-hydroxyvitamin D (25OHD), preferentially with a standardized assay. Infusion of calcium and phosphate in vitamin D-deficient rats results in normal mineralization of hypertrophic cartilage and bone [4]. In systemic VDR knock-out model, rickets and osteomalacia can be prevented by a diet rich in calcium and phosphate with lactose supplements to improve intestinal calcium absorption [1, 5]. By promoting optimal extra-cellular calcium and phosphate concentrations, the vitamin D system ensures the mineralization of newly deposited bone and cartilage matrix [6]. In a recent report, an infant with hypophosphatasia suffered vitamin D deficiency-induced rickets, although serum calcium and phosphate were always entirely normal [7]. However, rickets was cured by vitamin D treatment alone, confirming some in vitro data which indicate a direct effect of vitamin D on osteoblast mediated mineralization [8]. These findings indicate that optimal extracellular calcium and phosphate concentrations are necessary for cartilage and bone mineralization, but vitamin D could exert a direct effect on the mineralization process as well, under certain circumstances.

The doses of cholecalciferol recommended for preventing rickets in childhood and osteomalacia in adults are widely accepted [9–11]. Indeed, a dose of 400 IU/day (10 µg) of vitamin D is recommended, together with 500 mg/day of dietary calcium [9] for the prevention of rickets. For the treatment of nutritional rickets, 2000 IU/day (50 µg) of vitamin D should be administered for at least 3 months, together with 500 mg/day of calcium. In adulthood, the various recommendations for the prevention of vitamin D deficiency range from 400 to 800 IU/day (10–20 µg) of cholecalciferol (reviewed in [10]). In the field of bone diseases, 800–1000 IU/day is a dose which is consistently recommended in the oldest old individuals [12–14], whilst doses lower than 700 IU/day appear to be ineffective on fracture risk [15]. Reduction of hip fracture risk is achieved by combining daily vitamin D and calcium [16]. In the most recent meta-analysis, the association vitamin D and calcium was associated with a 6 and 16% reduction of any fracture and hip fracture, respectively [17]. In this analysis, five out of six included randomized controlled trials were using 800 IU/day. With this dose, 25OHD levels reach the threshold for sufficiency of 50 nmol/l [18].

In several countries, calcifediol (25-hydroxyvitamin D) is often prescribed for the prevention and/or treatment of vitamin D deficiency. Calcifediol appears to display a higher rate of intestinal absorption as compared with cholecalciferol. This compound could be particularly useful in liver failure, in drug-induced alterations of liver cytochrome enzymes activity, in genetic disorders of 25-hydroxylase and in gastrointestinal diseases [19]. Based on 25OHD circulating values, it appears that there is an at least threefold higher potency of calcifediol as compared with cholecalciferol [20], despite an important interindividual variation. Under these conditions, 10 µg/day of calcifediol may be equivalent to 1200 IU/day of cholecalciferol and could be considered as a safe dose. No hypercalcemia was recorded with 20 µg/day of calcifediol [21].

## Upper limit of safety

The upper limit of safety of cholecalciferol is proposed to be 4000 IU/day [11]. This upper limit of tolerance is based on 25OHD determinations and on the risk of hypercalcemia occurrence [18, 22–24]. A chronic dose of 95 µg/day (3800 IU/day) was considered as the lowest dose causing hypercalcemia in healthy adults. Indeed, elevated serum calcium was found in six subjects consuming 95 µg/day for 3 months [25]. In contrast, 100 µg/day (4000 IU) were compared to 25 µg/day (1000 IU) in a 5-month trial [23]. The maximum plateau serum 25OHD was 120 and 100 nmol/l, respectively. Neither serum calcium nor calcium-to-creatinine ratio in second morning void significantly changed during the study. In a recent trial including 373 62-year old healthy and vitamin D-replete subjects, 400, 4000 and 10,000 IU were administered daily for 3 years [26]. Hypercalcemia (total serum calcium > 2.55 mmol/l) occurred in 0, 3 and 9% in the 400, 4000 and 10,000 IU/day groups, respectively. A 24-h urinary excretion higher than 7.5 mmol/day, which defined hypercalciuria, was detected in 17, 22 and 31% of the corresponding groups.

Vitamin D overdosing leading to overt hypercalcemia is rare and can result from the endogenous overproduction of the active metabolite calcitriol through 1-alpha hydroxylation in abnormal macrophages as encountered in sarcoidosis or other granulomatosis, or through vitamin D release from fat tissue storage in case of rapid loss of fat mass [27]. However, the most frequent cause of vitamin D overdosing is exogenous, i.e., by excessive intakes. Vitamin D excess due to iatrogenic administration of pharmacological doses of vitamin D is a rare cause of hypecalcemia. Large doses used to be given in the treatment of hypoparathyroidism before the availability of active vitamin D metabolites. Vitamin D excess is associated with increased intestinal calcium absorption and bone resorption [28]. The latter is responsive to bone resorption inhibitors. Because of the prolonged half-life of the metabolite 25OHD, hypercalcemic-hypercalciuric syndrome can persist for several weeks to months after treatment discontinuation, with an important morbidity and even extensive and permanent soft tissues damages by mineral deposits. A meta-analysis has included 37 randomized controlled trials of vitamin D supplementation in which occurrence of hypercalcemia was recorded and 14 for hypercalciuria [29]. It turned out that long-term vitamin D supplementation was associated with an increased risk of hypercalcemia and/or hypercalciuria (relative risk 1.54 and 1.64, respectively. Hypercalciuria was only detected in trials with vitamin D doses superior to 800 IU/day.

Clinical expression of vitamin D overdosing includes hypercalcemia, hypercalciuria, and mineral deposits in soft tissues. Over the last few years, the safety of 4000 IU/day, which is consistently considered as the upper limit of safety, has been challenged, since the risk of adverse events may depend not only on the dose, but on the outcome, the treatment regimen, and possibly the age, sex and vitamin D status. The therapeutic window may be narrower than hitherto accepted for bone health, fall risk, frailty, kidney stones and mortality.

## Bone health

In a randomized controlled trial, 400, 4000 or 10,000 IU/day of vitamin D were administered to 311 healthy vitamin D-replete, non-osteoporotic, 62-year old subjects [30]. At the end of 3 years, changes in distal radius volumetric BMD, as assessed by high resolution peripheral QCT, were − 1.2, − 2.4 and − 3.5% in the 400, 4000 and 10,000 IU/day groups, respectively. The values in the two latter groups were significantly lower than in the 400 IU group. At distal tibia, volumetric BMD was lower than the 400 IU group in the 10,000 IU group only. There was no difference in total hip areal BMD. Serum 25OHD above 125 nmol/l was associated with accelerated bone loss [30]. While 100,000 IU/months corresponding to 3300 IU/day of vitamin D was without any effect on BMD nor fracture risk in individuals without vitamin D insufficiency (VIDA study) [31], there was a 2.6% increase in spine BMD in a subset of patients with 25OHD less than 50 nmol/l at baseline [32]. In another trial conducted in subjects with a normal vitamin D status (mean baseline 25OHD: 77 nmol/l), vitamin D supplements of 2000 IU/day of cholecalciferol did not influence areal BMD at the spine, hip or whole body level over 2 years (VITAL study) [33]. When given to patients with 25OHD levels < 50 nmol/l, 2800 IU/day improved distal tibia bone strength, but not axial skeleton areal BMD over 3 months [34]. Not only high dose of vitamin D, but also the regimen may be associated with some adverse effects, as demonstrated by a higher fracture risk when 500,000 IU of cholecalciferol (equivalent to approximately 1400 IU/day) vitamin D was given on a yearly basis [35]. This study included 2256 women older than 70 years. The fracture risk increased by 26% and was mostly observed during the first 3 months after dosing. Such a higher fracture risk is in agreement with 300,000 IU (equivalent to 820 IU/day) given intramuscularly once a year, which was associated with a 49% increase in hip fracture risk [36]. In neither studies, subjects were recruited on the basis of low 25OHD levels. In another trial, the same total 300,000 IU annual dose of cholecalciferol was administered as 100,000 IU at 4-month interval [37]. In this trial, there was a 22% reduction in fracture risk. This indicates that the regimen (yearly vs 4 monthly) rather than the dose determines the outcome. As potential mechanism, should be reminded that a single oral bolus of 600,000 IU vitamin D was associated with a 50% increase in sCTX, which lasted for at least 2 months [38].

## Falls

Like for fracture risk, a yearly oral administration of 500,000 IU vitamin D of cholecalciferol (equivalent to 1400 IU/day) was harmful since it was associated with a 15% higher risk of falling [35]. Monthly doses of 100,000 IU in vitamin D-replete individuals with a mean age of 66 years were without any effect on fall risk [31] over 4 years. The same monthly dose given over 12 months to long-term care residents, with a mean age of 81 years and 25OHD lower than 50 nmol/l in a third of them, reduced acute respiratory incidence by 40%, but was associated with a more than twofold higher rate of falls, when compared to a 400–1000 IU/day standard dose [39]. This indicates that the efficacy or the potential toxicity of vitamin D supplementation depends not only on the baseline vitamin D status, but also and most importantly on the type of variable assessed. A possible U-shape curve, with a narrow optimal therapeutic window, in the relationship between fall risk and vitamin D dose, was suggested by the higher incidence of fallers with 60,000 IU monthly (corresponding to 2000 IU/day) as compared with 24,000 IU (i.e., 800 IU/day) in a one-year trial [40]. When 300 µg/month (equivalent to 10 µg/day) of calcifediol was given in addition to the lower dose of cholecalciferol, falls were as frequent as with the higher cholecalciferol dose, supporting an equivalence of 10 µg calcifediol and 1200 IU cholecalciferol. In this trial, there was no difference in the primary endpoint, i.e., changes in short physical performance battery, between both groups. Paradoxically, the patients who reached the highest quartile of 25OHD (> 110 nmol/l) by 12 months displayed a more than fivefold higher risk of falling than those reaching the lowest quartile (< 75 nmol/l). In a 3-month randomized, double blind, controlled trial, a dose of 2800 IU/day was given to women 60–80 years of age, with a baseline 25OHD lower than 50 nmol/l. It reduced maximal grip strength (− 9%) and knee flexion strength (− 13%), and increased by 4.4% the timed up and go test [41]. In another randomized controlled trial, the effects of a wide range of daily vitamin D doses on falls was tested in 66-year old women with baseline 25OHD levels lower than 50 nmole/l over 12 months [42]. There was a significant decrease in falls with medium doses of 1600, 2400 and 3200 IU/day. In contrast, with the high doses of 4′000 and 4800 IU/day, fall rates were as high as in the placebo group and significantly higher than with the medium doses. Fall rates were higher as serum 25OHD exceeded 100 nmol/l. The relationship appeared to slightly differ in African-Americans, since a higher fall incidence rate was not observed the 4000–4800 IU/day doses. The conclusion of this study was that the tolerable upper limit of 4000 IU/day may be lower in elderly women, particularly in those with a fall history. In the same study, areal BMD did not change irrespective of the dose [43]. Along this line, postural sway was not affected between daily doses of vitamin D of 400, 4000 and 10,000 [44], vitamin D daily equivalent of 5700 IU was not different from 800 IU in terms of muscle strength and balance [45], changes in muscle strength and mass were not different in the 40,000 IU/week vitamin D2 (equivalent to 5700 IU/day) and placebo groups [46], whilst 1000 IU/day increased lower limb muscle strength [47]. In a systematic review and meta-analysis published in 2011, the conclusion was that daily doses of 800 to 1000 IU consistently demonstrated beneficial effects on muscle strength and balance [48]. In a 2014 meta-analysis, vitamin D increased muscle strength mainly in subjects with baseline 25OHD less than 30 nmol/l, and tended to be more effective in the ≥ 65 years population [49]. However, because of the large heterogeneity in the supplementation protocols, it was not possible to detect any dose–response relationship.

## Frailty

In the Study of Osteoporotic Fracture, a U-shape relationship between frailty and circulating 25OHD levels has been reported in women [50], but not in men [51] at baseline. Such an association in a large cohort may suggest some harmful influence of high vitamin D levels, at least in one gender. Probably in relation with vitamin D binding protein, which is higher in females than in males, particularly before menopause, in pregnancy and in hormone oral contraceptive users, total 25OHD might be slightly higher in females than in males, but without evidence of a difference in the free part of the metabolite [52]. This effect could be mitigated by the higher body fat in women. However, in the absence of randomized controlled trial assessing the effects of various doses of vitamin D on frailty prevalence, a confounding by indication phenomenon cannot be excluded. Indeed, in a study conducted in nursing home veterans, the more likely to be frail were the groups of non-users of vitamin D supplements with low 25OHD levels, and those receiving vitamin D supplements and having high 25OHD levels, as compared with vitamin D supplements non-users, but with similar high 25OHD levels [53].

## Renal stone

In the WHI trial, a 17% higher risk of renal stones has been reported [54]. With a mean baseline calcium intake of about 1150 mg/day, the subjects were randomly assigned to 400 IU/day of vitamin D and 1000 mg/day of elemental calcium, or a placebo. The higher risk of renal stones is more likely attributable to the high calcium intakes than to the low vitamin D dose. Indeed, a meta-analysis including nine trials and 9619 patients, but not the WHI study, the relative risk of developing renal stones was 0.66 (not significant) for vitamin D doses ranging from 800 to 5700 IU/day (8 out of 9 studies used vitamin doses below 2900 IU/day) [29]. Surprisingly, in a dose–response study, with vitamin D supplements ranging from 400 IU to 4800 IU/day for 12 months, the prevalence of hypercalcemia or hypercalciuria did not appear to be dose-dependent [55]. Over a median observation time of 3.3 years, monthly supplementation with 100,000 IU (equivalent to 3300 IU daily) did not influence the incidence of renal stones or hypercalcemia episodes [56].

## Mortality

Numerous observational studies have shown a higher all-cause mortality with vitamin D deficiency/insufficiency, on a 25OHD concentration-dependent manner [57–61]. Below 30 nmole/l, mortality was increased more than twofold. A nadir in the curves was found at a 25OHD level of around 75 nmol/l. Some trend to higher mortality could be suggested above 120 nmole/l [57, 61, 62]. However, this trend disappeared in the NHANES III study when 25OHD was again measured using a standardized assay [62]. Vitamin D3, but not vitamin D2 nor vitamin D active metabolites supplementation was associated with a lower mortality [59, 60]. Vitamin D with calcium reduced mortality by 6% in a patient level pooled analysis of 70,528 patients from 8 vitamin trials [63]. No randomized controlled trial had mortality as primary endpoint. Mortality was mainly significantly decreased when mean baseline 25OHD was below 50 nmol/l [60]. In contrast, 100,000 IU vitamin D2 given at 3-month interval was associated with 19.7% deaths as compared with 16.5% in controls, in 85-year old subjects living in care home accommodation [64]. There was no difference in mortality when 54-year old patients with heart failure were given a dose as high as 4000 IU/day vitamin D for 3 years [65].

## Conclusion

The upper limit of vitamin D dose safety may differ depending on the vitamin D status of the recipient (vitamin D deficient, insufficient or sufficient), the dose, the regimen (daily or intermittent administration) and the outcome (falls, hypercalcemia, hypercalciuria). Age and sex may also play a role. The prevention and/or correction of vitamin D deficiency/insufficiency with 800–1000 IU/daily of vitamin D are safe, as would be 10 µg/day of calcifediol. Because of their potential harm, larger doses given on the long term or in intermittent regimens should not be selected.
